# Psychological Quarterly Retrospect

**Published:** 1859-01-01

**Authors:** 


### *Psychological Quarterly Retrospect.*

There are few psychical phenomena of greater interest than the recrudescence of popular delusions. In our last Quarterly Retrospect we had occasion to notice one of several instances of belief in the old doctrines of witchcraft, which had been manifested in this country during the last quarter; and we could, if it were necessary, adduce other instances which have occurred during the present quarter. We recur to the subject, however, mainly in consequence of a remarkable outbreak of witchcraft superstition in Sweden, of which the following account is given in the *Athenæum* (October 30th, 1858) :—

"The mysterious phenomena of superstition, which have ever been peculiar to the Scandinavian countries, as a thousand examples in history may prove, seem to rise again, and to glare mockingly in the face of the nineteenth century. A strange and gloomy tale is reported from the Swedish valley countries (*Dalarne*). The prebendary, Dr. Hvasser, in Leksand, has received orders from his Chapter to inquire into the superstition and witchcraft-nuisance at Gagnef and Mokfjärds Annexen, in the Swedish valleys. The old Blackulla Journeys have risen from their centenary grave, and begin to haunt again the Delar-neighbourhood. Again the charmed horn is seized, and with the swiftness of lightning the Journey goes up the church-steeple, and from there to a mysterious place, where an alliance is made with the Prince of Darkness, who, with a pen dipped in the blood of the little finger, writes the name of the poor wretch into his book—exactly as at the time when much less was sufficient to cause one to be burnt alive. Yet there is some difference. Blakulla (the Brocken or Blocksberg in the Harz Mountains) is not named; the place in question is called Josephsdal, near Stockholm! The Journey is performed in the following way:—The child that is to go on it is first transformed, inside the room, into a worm; as such creeps out of a hole of the window; then takes the shape of a magpie, and then turns at last into a child again. Now it mounts up the church-steeple on a calf's or cow's skin. But here we have a variation again from the old practice, which was, to scrape some metal from the bells, repeating at the same time these words:—'May my soul never come into God's kingdom before this metal is joined with the bell again.' The children now-a-days content themselves with taking some flour to Josephsdal for the preparation of the 'Welling'—a mysterious dish eaten at the banquet. Satan is there called Nors or Norsgubbe (Gubbe meaning the Old One). He is said to wear shaggy boots, which he sometimes, when the scene becomes more animated, flings from his legs. With the exception of a few women, it is especially children who must talk of their journeys to Josephsdal, and of their alliance with Norsgubbe. The greatest part of the children in the parish of Mokfjärds Annexen (from 50 to 100 in number) has caught this strange disease of the mind, and some give a minute account of a great many queer circumstances of their journey, and the banquet at Josephsdal. Yet these uncouth fancies do not seem to affect in the least the health of the children; they are well, and seem perfectly happy. Not so the parents, who are in a state of deep despondency at the thought of their children having thus fallen into the clutches of Satan. Those children who are innocent of these horrible illusions, but who have been denounced by the others, nevertheless, as travelling companions, are tormented and tortured by their benighted parents to extort confession. Thus, for instance, a little boy, Grabo Pehr, who has several times been at Josephsdal, denounces a little girl to her mother, as having been with him at Josephsdal, and, in order to give strength to his assertions, he tells the superstitious mother that at the banquet some of the warm 'Welling' had been spattered into her daughter's face, and that this was the reason why her open wound would not heal. The child, in fact, had a bad wound close to her eye, which remained sore and swelling, thus apparently confirming the boy's accusation in the frightened mother's mind. However, the poor child knew of no Josephsdal, and no warm 'Welling,' and, consequently, could not be brought to confess. Fortunately, the excitement among the children has now begun to subside in the 'Dalarne,' and, it is to be hoped, will soon be over altogether in that neighbourhood. But, as in the case of other epidemic diseases, this psychical disorder seems to spread, and symptoms have shown themselves in the neighbouring parishes. The dejection of the elder part of the inhabitants seems still very great; a gloomy cloud has spread over the face of the country, and it may not pass over so soon from the minds of the afflicted parents."

In this outbreak of the belief in witchcraft, as well as in the recent instances of the belief which have happened in England, there have been reproduced, with barely any variation, the notions which were prevalent regarding witchcraft in the fourteenth, fifteenth, and sixteenth centuries, and which have been handed down traditionally among the more ignorant classes of our own and of the Swedish population. Apart from the interest which attaches to the manifestation about the same period of the same superstitious delusion in two different countries, which have but slight bonds of connexion, the psychical phenomena which are witnessed during the progress and development of the delusion, are of value as furnishing data for the further examination of the physical and psychical conditions of superstition—a problem very far from being solved, and which may, perhaps, be most successfully dealt with by the examination of instances of a well-defined superstition, under circumstances so unfavourable to the development of superstition as those which are commonly believed to exist in our own country.

Moreover, the occurrence of superstitious delusions is useful as an index of the mental standard of the class of persons among whom they happen, and they form no contemptible gauges by which to measure the success which may have followed any efforts for the mental cultivation of a community. Usually, the popular superstitions which still exist in this country are found to differ from their pristine form, and to have been modified by the changed and changing social conditions of the period. Goethe well understood this liability of superstitions to change with the differing circumstances of different periods, when, in "Faust," he represented Mephistopheles as saying,—

"Refinement, too, which smoothens all
O'er which it in this world has pass'd,
Has been extended in its call,
And reached the devil, too, at last.
That northern phantom found no more can be,
Horns, tail, and claws we now no longer see;
As for the foot—I cannot spare it,
But were I openly to wear it,
It might do greater harm than good
To me among the multitude.
And so like many a youth besides,
Who bravely to the eye appears,
Yet something still contrives to hide,
I've worn false calves for many years

To what extent the active elements of superstition still affect society in England cannot easily be said; but we suspect that, under other names and different forms, they are much more prevalent and energetically at work than is commonly supposed. Not that we regard this state of affairs (assuming that it exists) to be indicative of the failure of those efforts which have been made, by education, science, and art, to secure the mental advancement of the nation. So far from indulging this opinion, we consider the changed forms in which the active elements of superstition commonly manifest themselves to be certain proofs of the beneficial influence and of the success of those measures, notwithstanding that that success may fall considerably short of Utopian dreams of perfection. Others, however, who witness from time to time the up-heavings of what have been thought to be extinct superstitions; who see, also, the vigorous persistence of crime and immorality in every form; and who cannot, or will not, comprehend the difference between the absolute prevalence of crime and immorality as arising from excess, and their apparent prevalence as arising from the greater light which is thrown upon them, and from the consequent greater distinction of the points of demarcation between vice and virtue, are apt to put little faith in the intellectual advancement of the age. To such doubters we would commend Lord Brougham's account of the class of works which found favour with the English public before the rise of popular literature, properly so called; and, also, his statement of the influence which this form of literature (one only of the agents employed to quicken and improve the intellect) has exercised upon the mental and moral state of the community.

In an address upon *Popular Literature*, delivered at Liverpool during the meeting of the National Association for the Promotion of Social Science, his Lordship said:—

"A survey of the moral world, individual and social, is fitted to raise the same emotions which inspire us in casting our eyes over the solar system; the movements, the proportions, the action of attractive, repulsive, and disturbing forces, all at first sight little to be comprehended, and apparently without arrangement or plan, but all, when deeply considered and carefully compared, reducible to certain rules, and fixed unchangeable order. As new bodies are discovered in the heavens by marking their mutual attraction with those before known, the attractions of science and of letters will disclose to the just and learned observer genius already existing, but now first drawn forth to the view. But the *nebulæ* occupy the largest space in both firmaments; and their moral importance is unspeakably superior to that of either the more shining or the greater luminaries. The benefits are beyond all price which the bulk of the community derive from the influence, and from the assistance of Popular Literature; nor can anything be more unreflecting than the doubts which have been raised of its beneficial tendency.

"We may begin with the broad fact of the harmless character, to say the very least, of the amusement which it affords. While we admit it to be certain that a considerable portion of these works is devoted chiefly to entertainment, this is certainly of an altogether innocent kind. But it has come in the place of a different class of publications. When Mr. Hill proposed the *Penny Magazine*, the first of the kind now so happily established in the confidence of the people, Charles Knight (a great public benefactor both as an author and publisher) brought him a list of no less than nine weekly papers devoted to the circulation of the most abominable matter; morally scandalous and obscene; religiously not simply infidel, but scoffing and ribald; politically preaching anarchy, hardly even confined to the crazy dreams of socialism, but as if the editor were that boy become a man, who, when the Sovereign went to meet his parliament, had been arrested for bawling out, 'No king! no church! no lords! no commons! no nothing!' The *Penny Magazine* drove these vile publications absolutely out of existence. A most feeble progeny alone was left to succeed them; it skulked in corners, and ever since has scarcely been heard of. It was like the effect of the Society's *Almanac*, which put an end to the disreputable fortune-telling tracts before published by the Stationers' Company, and abandoned by them, other and rational year-books being substituted in their place, perhaps immediately, certainly as soon as the illustrious statesman and warrior at the head of the Government, without any application on our part, gave directions that the Society's *Almanac* should be used at all the public offices. But it is not only irreligious, immoral, and fraudulent publications that have thus been supplanted; the far less hurtful, yet by no means commendable works which study to give the mere excitement of horror by dealing in accounts of brutal murders and cruel seductions, and romances abounding in such descriptions, together with ghost stories—these, once so greedily pored over, now find but little acceptation, and have ceased to be in demand. It is most satisfactory to find that the natural preference of the people is for the better kind of writings. At times of political or religious excitement those of a worse cast may have some success, but it is temporary. The works of Carlile and Paine have long ceased to attract readers, the people falling back upon papers which combine harmless recreation with some instruction; and the tendency of public prosecutions to give them an interest which they had not naturally was found so manifest, that the Government has long taken the safer course of letting them alone.

"But it would be a great mistake to suppose that the benefits of the Popular press are negative only. The tales composed for the working-men's hours of relaxation are of a kind that address themselves both to the understanding and the heart—at once giving lessons of instruction and fostering the kindly affections. Nor can anything be more groundless than the charges that have been brought against them. Two of these may be at once stated and disposed of. First, we are told that the stories given relate to persons and scenes in high life, and that none other interest the working-classes. This is entirely contrary to the fact. That these classes wish occasionally to know what passes among their superiors is quite true, yet not more true than that their superiors desire to dwell upon the actions and the sufferings of each other; but what most powerfully excites the humbler classes, and most deeply imprints itself on their memory is the story of the actions and the sufferings, the good and evil fortune of their brethren and equals. They delight to dwell on the struggles of heroism, the endurance of privation, the agonies of anxiety, the resignation under sorrow, of the humbler classes, their own brothers and sisters. He who, vividly, above all feelingly, portrays a noble heart throbbing under a fustian jacket or a cotton gown, records the tears shed for the untimely loss of the young, or the removal of the protection made habitual and venerable by length of years, is sure to find eager and sympathizing readers. Nor will he less awaken their minds, though to emotions of a different kind, who describes the anxious fears of conscious but undetected guilt, the ever-wakeful remorse when discovery is not dreaded, and the worthlessness to secure happiness of vicious, though successful courses. Characters thus taken from humble life, and scenes laid in its haunts, most strongly rivet the attention of the working-men and their families. And wherefore this? Because the case may be their own. The fiction of to-day may to-morrow be the sad or the happy reality of their own lot. That the narratives and the descriptions which thus attract and thus move them, are fitted to affect others as well may be safely affirmed. It is from experience, no less than from the relation of friends in the higher classes, that we may describe it as impossible to read some of these stories with a dry eye. It must not, however, be supposed that none of the romances, favourites of the great, are thus made conducive to the entertainment of the poor. Some of Sir W. Scott's have been given in these publications; and it is only to be wished that they had been accompanied with warnings against the perversions of history, as well as the false and indeed obsolete political opinions in which some of that great writer's tales abound.

"But next it is alleged that what is termed the new—it should rather be called the improved—literature of the people supplants more solid and more useful works, and the multitude of the readers is given as a proof of this. It is assumed—most falsely assumed—that these are withdrawn from the perusal of other publications. On the very contrary, they are added to the body of former readers, and their numbers prove it to demonstration. Take three instances—*Cassell's Illustrated Family Paper* began this year with a sale of 300,000, and the *Family Herald* issues 260,000. The *London Journal* is asserted to have a circulation of 500,000; but its present actual sale is from 320,000 to 350,000. What papers and other periodical works did these 800,000 purchasers take, and what did the 2,000,000 who in the whole peruse these three papers read before they were brought out? It is quite manifest that this is, if not wholly, yet in a very great proportion, a clear addition to the number of persons who formerly saved from their earnings a penny weekly, and laid it out in purchasing what would help them to pass an hour or two of rest, without the wearied sense of unoccupied time, or the pernicious resource of drinking. The provision is only made for such as before had none. A new food has been presented to the mind. They who fancy that it comes in the place of other and more wholesome fare would have objected to the potato being cultivated, because it lessened the gains from the growth of wheat; whereas it only produced a supply for those who else were doomed to starve, or to linger out a feeble life on most scanty diet. Nay, the objectors may peradventure belong to the class which would not have the resource of foreign markets opened to us, lest the security against a famine, by giving our people access to the produce of other soils and other climates, should lessen the numbers who consume that of our own. There used to be some persons, nay, at one time no small number, who thus held and thus felt alarmed. The race is supposed to be long extinct; and specimens of it are only to be found preserved in the antiquary's collections of political curiosities, as the fossil remains of long-lost animals which once peopled our globe may be seen in the museum of the geologist.

"It is quite as great a delusion under which those labour who figure to themselves the promoters of Popular Literature as indifferent to the encouragement of more severe studies, and the cultivation of profounder science. We of the Useful Knowledge Society can well recollect that exactly the same prejudice prevailed, or if it did not, was dishonestly sought to be raised, against the preparation of scientific works in a cheap form, and designed to give information of the most solid and even profound description. Some of the very persons who were remunerated, and amply remunerated, for their writings, derided what they called 'sixpenny science,' because a treatise once a fortnight for several years was published at that price; but by whom composed? By such mathematicians as Professor de Morgan, such natural philosophers as Sir David Brewster, both a discoverer and a teacher, such botanists as Professor Lindley. It was plain enough that some of those who thus complained of the treatises as not profound, could not have read one line of them, from their own profound ignorance of the subjects. Contemporary with the *Penny Magazine* was the *Penny Cyclopædia*, of which it is enough to say, that so accomplished a scholar as Professor Long being the conductor, no less a mathematician than the Astronomer Royal has published in a separate form his valuable contributions to the work; papers, too, composed in so plain and popular a manner as to bring the most sublime truths of the Newtonian philosophy within the comprehension of readers very moderately acquainted with the mathematics."

If we would have other information respecting the beneficial influence of mental cultivation on the social condition of the nation, we may find it in the statistics of crime. Mr. Neison, than whom no higher authority exists, states, as the results of his researches into the relation of education to crime, that—

"By adopting the test of education or instruction furnished by the marriage registers of the country, and further analysing the groups referred to in the preceding paragraphs, by dividing each into two sections—by the one of which will be represented the population of highest education, and the other the population of lowest education:—in fact, so analysing the various districts and groups of counties, that they differ in respect of education only,—it is found that, out of the 22 different combinations formed of the various districts in England and Wales, in every instance there is an excess of crime where there is the least education or instruction; and, comparing the respective sections of each group of counties, it will be seen that there is an average excess of 25 per cent. of crime in the sections of inferior education over that of higher education, and in some districts the excess is as much as 44 per cent.

"That it is hence obvious that the very small amount of education, or rather instruction, implied by the test here adopted, has a powerful influence on the criminal calendar of the country, and that the introduction of this further element into the investigation of the relative amount of crime, removes many anomalies not otherwise to be understood."*

Under the existing state of things in Ireland, where agrarian outrage has once more become common, and illegal societies have

* "Contributions and Vital Statistics," by G. P. Neison, 1858, p. 405.

grown again into being and activity, it would be well to ask what part in the causation of these evils ignorance alone might play, apart from peculiarities of race and asserted social wrongs. The *Times* of Nov. 20th contains the following suggestive paragraph:—

"A correspondent of *Saunders's Newsletter* supplies some curious statistics in connexion with the march of education in the Gweedore district. The writer says:—

"'I have now lying before me a copy of the *Warder* of the 13th inst., in which the proceedings, papers, and resolutions bearing on the attempted assassination of the Rev. Alexander Nixon are fully set out. Among those I find the names of 94 tenants—of course the principal men, who in their own persons, and representing the tenantry not only of their own, but also of the neighbouring townlands, amounting to more than 1500 persons—uniting in a petition to the Executive, praying a remission of taxation imposed on the district for lawless proceedings, expressing great contrition for the same, promising amendment for the future, and confessing that the allegation of destitution which was lately presented to Parliament was devoid of foundation. Having read all this fine composition, will it be credited in the 19th century—an epoch of diffused knowledge—will the *Times* believe this astounding fact—that there was not a single man among the 94 subscribers who could write his name; and it may be safely inferred that, had there been one among the remaining 1,500 who could, he would have been called on to perform the important part of writing his name? Will the English people believe this fact, after squandering such immense sums for education? What else can arise than murder, robbery, and the total disorganization of the social system, from such a horrible state? Will the Executive institute a searching inquiry into the cause of this frightful destitution of the common rudiments of education?'"

To those who persist in believing that the period in which they live shows a marked deterioration from that immediately preceding, and who regret the decadence of those social conditions when the chivalrous Dick Turpin was the type of the highwayman, and the refined Jack Sheppard the type of the burglar, it must be refreshing to find that a heightened sentiment of the proper respectability of a criminal calling is not altogether extinct among the criminal class. The following example will show this:—

WORSHIP-STREET.—Thomas Jordan, John O'Brien, and Henry Merritt, three well-dressed young fellows of 21, all well-known thieves, were charged before Mr. D'Eyncourt under somewhat peculiar circumstances.

Deeble, a detective of the H division, said,—I was on duty in plain clothes last night in Commercial-street, Whitechapel, when I saw Merritt lift up the tail of a gentleman's coat and thrust his hand into the pocket; he withdrew his hand without stealing anything, though I distinctly saw the gentleman's handkerchief in his pocket; Jordan and O'Brien were "covering" Merritt while so acting. I knew them all to be regular thieves, and being too well acquainted with their character to attempt taking them alone, I followed them till I saw them enter a public-house, which is notoriously the resort of such customers, and then I got assistance and took the three.

O'Brien (contemptuously).—Took the three. Yes, and you brought five officers with you to do it. Why didn't you take us at once?—we offered no resistance.

Deeble.—No, you offered no resistance because you knew it would have been useless; I knew, too, that the house was likely to have more of you in it, and when I got in I found I was right, for there were 12 of you there.

Merritt.—Why, sir, I have just come out of the hospital, and had not the slightest idea of such a thing.

Jordan.—Nor I neither, I'm sure; we were certainly together, but that was all.

O'Brien (to the magistrate).—Now, sir, I ask you—and I ask you because you must know—is it at all feasible that we three should try to steal a paltry handkerchief? It's a perfect insult to suppose so, and I am really ashamed to stand here charged with such an offence.

Mr. D'Eyncourt.—You mean because it is so paltry?

O'Brien.—Just so; it is paltry. As far as I am concerned myself, I own that, for my part, I should not care if the charge was for anything worth while, but this is for a paltry handkerchief. Why, look now, sir, any one can always buy a silk one for eighteenpence, and this one was only cotton; is it likely?

Mr. D'Eyncourt.—Does any one else know the prisoners?

Serjeant, 12 H.—I do, your worship; I know them all to be regular thieves.

Deeble.—And I know also that O'Brien has been charged before.

O'Brien.—Well, I own to that, certainly; but I have been at work since, and I can prove it.

Mr. D'Eyncourt.—You will each of you go for three months' hard labour in the House of Correction.

O'Brien.—I am very much hurt to hear you say that, your worship. Am I to understand that you send us for stealing this handkerchief?

Mr. D'Eyncourt.—No, no, not for actual stealing; for the attempt to do so.

O'Brien.—Oh, ay, that's quite right; thank your worship; I'm sorry I troubled you.—*Times*, Oct. 27.

Do we not hear the shade of Pistol whispering, "Convey, the wise it call: steal! Foh; a fico for the phrase!"?

In our last Quarterly Retrospect, when speaking of the crime of the quarter, we stated our intention of considering the horrible murder at Darley, in Yorkshire, after the trial of the murderer had taken place. The trial has been recently held, and the prisoner acquitted on the ground of Insanity; but the importance of the whole case in a medico-legal point of view is such, that we have given a full report of the trial elsewhere in the present number of the *Journal*.

Among the criminal events of this quarter, at home and abroad, there are two which merit consideration. Both are remarkable for the ages of the principal actors in them. The first of the events, in order of time, occurred in New York, and it is thus reported in the *New York Weekly Times* (October 30) :—

At a late hour on Tuesday night a more horrible tragedy was enacted in this city than we have ever before had occasion to record. Francis A. Gouldy, aged nineteen years, attempted at his father's house, No. 217, West Thirtieth-street, between Eighth and Ninth Avenues, to murder his father, mother, two brothers, and two servant girls, and terminated the terrific scene by effectually blowing out his own brains. Although the wounds inflicted on his father, brothers, and one of the servant girls, are of such a fearful nature as to preclude any hope of their recovery, none of them had expired at the time of our going to press.

Young Gouldy entered Showler's lager-bier and oyster saloon, between Twenty-eighth and Twenty-ninth streets, Eighth-avenue, about nine o'clock on Tuesday night. He was accompanied by a boy thirteen or fourteen years old. They partook of oyster stews, but drank no liquor. Gouldy was in the habit of frequenting this saloon, but for a long time past had drank no intoxicating liquor. On Tuesday night he left this saloon a little after half-past nine o'clock, in a perfectly sober state. While there, he remarked that he had had a dispute with his father about money. From the fact that he reached home about twenty minutes before ten o'clock, and that the distance from the saloon to his father's house is not over five minutes' walk, he could not have stopped at any place on the way. He rang the door bell, his father having refused him a night key. The door was not opened by a servant, but by his father, who was awaiting his arrival. Immediately on his entrance, the old gentleman charged him with abstracting a Savings' Bank book from his private desk, and procuring money thereon, representing the act to be as bad as a direct robbery. The young man replied that as the account at the bank was opened in his name, he had a right to take the book. Soon after young Gouldy retired.

It would seem that he proceeded to his room in the rear of the house, on the third floor, and in a cool and collected manner, changed his dress, removing his coat, vest and cravat, hanging his watch on a nail by the side of the mirror, taking off his boots, and even removing the sleeve-links of his shirt. Then taking a hatchet in his hand he descended the stairs in his stockings, without boots or slippers, entered the sitting-room where his father was just turning off the gas, and dealt him a blow on the head, fracturing his skull, and striking therefrom a portion of the bone from the temple, three inches long, and two and a-half inches in width. Mr. Gouldy fell, and the blood issuing from the fearful wound made a large and deep pool upon the floor. Mrs. Gouldy, who had just gone to bed, heard the heavy fall, and was in the act of raising herself to listen, when the son entered her room exclaiming, "Mother! oh, mother!" Instantly he seized her hand, and dealt her a severe blow upon the head with the hatchet, which deluged her face with blood. She screamed and sprang from the bed, when the young fellow repeated the blows until she was rendered senseless. He then repaired to the hall bedroom, where his two brothers, Nathaniel and Charles, aged thirteen and six years, were sleeping in the same bed. He struck at them both with the same weapon, cutting from the head of the eldest a piece of bone, two inches long and nearly an inch wide. The younger was not so severely injured, but his skull was fractured, and pieces of the bone chipped away.

The assassin next proceeded to the hall of the third floor, where the two servant girls, who had heard the noise, had come from their room, to listen. He immediately attacked them with the hatchet, dealing them frightful blows on the head. One of them wrested the weapon from his grasp, but he recovered it, and struck her to the floor, by a powerful blow. His sister Mary, hearing the struggle and screams of the servants, opened the door of her room, and saw the girls covered with blood, but did not recognize her brother. Supposing him to be a burglar, she retreated into the room, locked the door, threw open the window and shouted for the police. Some officers of the Ward (the Twentieth) hearing her cries, hurried to the spot, forced open the door, and were spectators of such a scene of horror as they had never before witnessed. The father lay upon the floor entirely unconscious, his face and head covered with blood. The mother was insensible in the next room, and also deluged in blood. In the hall-bedroom, the little boys were in a similar condition. Ascending the stairs, they found the servant girls lying in a pool of gore, entirely unconscious, while, in his own chamber, the wretched young man was stretched upon the floor, wallowing in his own blood, having committed suicide by shooting himself in the head with a pistol. His brains were scattered about the poor, and his right hand still grasped the pistol, two barrels of which were still heavily loaded. When the officers entered he was not quite dead. He gave a few convulsive gasps, uttering no word, and expired. Doctors Harmon, Sewall, and others, living in the neighbourhood, were called in, and rendered all the assistance in their power.

The wound which the suicide inflicted on himself was on the right side of the head, immediately behind the ear, the skull being greatly shattered. A pool of blood, two feet in diameter, lay thick and moist around his head. The surgeon's probe and the discoloration of the left eye show that the ball lodged near the frontal bone, on the left side of the orbit. He fell by the side of a cabinet, between the door and the fire-place. His coat was thrown carelessly on a chair; his vest was hanging on a bedstead; his watch was hung on a nail; his cravat thrown on the floor; one boot was standing by his side, the other thrown under a table by the window, and the stockings on his feet were soaked with blood, showing that before he fell he must have trodden in his own gore. A more terrible sight than the corpse exhibited yesterday, even those who have "supped on horrors," have rarely seen.

The particulars of this most fearful tragedy are more fully detailed in the inquest taken before the Coroner's Jury.

The first witness examined was Mrs. Jane Gouldy, the step-mother of deceased. Her testimony was taken while she lay in bed suffering intensely from her wounds, and labouring under strong nervous excitement. She deposed:

Deceased was my step-son; since July last he has not been in any employment; he has been clerk at Sullivan & Hyatt's, hardware merchants, in Platt-street; he has been in the habit of coming home usually at ten o'clock at night; his father was very strict in this respect; deceased was at tea in his usual good health and spirits at six o'clock last evening; after tea he went out; soon after he had gone his father discovered that a bank-book for 50 dols., which had been in his desk, had been removed; I at once suspected deceased and spoke to my husband about it; I asked Mr. Gouldy if he had not given this book to deceased, and he answered that he had not; deceased returned to the house at ten minutes before ten last night; his father let him in; he was in very good humour; Mr. Gouldy asked him if he had opened his desk; Frank answered that he had; his father asked him why he took the book; Frank replied that as the money was placed in the bank in his name he had a right to take it and do what he pleased with it; his father reprimanded him for this, and he went out of the room laughing; it was not a pleasant laugh, but rather fiendish and exultant; Mr. Gouldy remarked it at the time; the dispute took place in the front room of the second floor; after deceased left the room I went immediately to my bed-room, in the rear of the front room, and went to bed, leaving my husband in the front room.

Q.—What next did you observe? A.—About a quarter of an hour after, I heard my husband fall, and immediately deceased rushed into my bed-room with an axe in his hand, exclaiming, "Mother! oh, mother!" I raised my hands as he approached the bed, and said, "What is the matter, Frank?" Deceased took hold of my hand and struck me on the head with the axe, cutting me severely; he then rushed out; my nurse shortly ran into my room, she was wounded, and almost covered with the blood which had flowed from her cuts; I then ran into my husband's room, and saw him lying on the floor wounded and insensible; my little son, Nattie, fourteen years of age, was kneeling over his father, kissing him; Nattie was also wounded in the head; I raised the window and called for help; some persons came to the front door, and I went down and let them in; in all, there were six of us wounded; I heard during the night that Frank was found dead in his own room; Johanna Murphy and Elizabeth Carr are the names of my servants.

Mary E. Gouldy, sister of the suicide, testified: I live with my father here was at home last night; I was in my room, which is the back entry bed-room on the third floor, when I heard screams of "murder," "Frank, don't kill my father," and I don't know what; I opened the door and saw my brother, who is dead, on the third story hall fighting with Johanna Murphy; some light was burning, I am not sure which; I was frightened, went into my room, shut the door and locked it; thought what I should do, and did not know; I heard other cries of distress, but could not distinguish the voice; it was a female voice; murder was in the cry; I cried too; I raised the window and cried for any one to come to assist; I came out of my room, in a little while, and came down stairs, and found that assistance had arrived; I found a good many in.

Q.—Did you see anything further? A.—I don't know; I was so frightened that I forgot what I saw after; I cannot say for certain that I heard the report of a pistol, but I think I did.

*To a Juror*—I heard conversation between my brother and the servant; I thought that thieves had got into the house, and that he was protecting her; I did not think that he was the cause of the difficulty; my little sister was not in the room with me; I recognise the hatchet exhibited as the one which my brother usually kept in a trunk in his room; he procured it at the hardware store where he was formerly employed; he often used it about the house; my brother always carried a pistol; the one exhibited I think I have seen before in a drawer in his room; the knife I do not remember to have seen.

Thomas Stephen Showler, residing at No. 358, Eighth-avenue, testified: I keep an oyster-saloon at the number mentioned; I knew the deceased, and have probably been acquainted with him about six months; the last time I saw him alive was last evening between nine and ten o'clock; I am sure it was after nine, but I cannot say the exact time; I saw nothing unusual in his manner to attract my attention.

Q.—Did he appear to have been drinking? A.—No, Sir; he drank nothing in my place: he only took a stew of oysters: I can volunteer some testimony as to his habits: he told a person in my presence some time ago that he was under obligations to his father not to drink anything; a boy I should judge to be about 13 or 14 years of age was with deceased in my place last night; from ten to fifteen minutes elapsed from the time deceased entered my saloon to the time when he left it; when he went out he bade me good night; I was not intimately acquainted with him; he had been in my place several times.

*To a Juror.*—He had not the hatchet nor any other weapon in his hands when he was in the saloon, that I saw; I think I should know the boy who accompanied him if I saw him dressed in the same way; he wore a short round-about; I do not think I would recognise the boy by his daguerreotype.

No other testimony was taken, and the Jury, without a moment's hesitation, returned a verdict of "Suicide by a pistol-shot wound, inflicted by himself."

The following account is given of the murderer:—

"Francis, who was the eldest son, and the author of this horrible tragedy, would have been nineteen years of age on the 19th of next April. At the common schools, while young, he made only tolerable progress. At fourteen he was sent to a boarding-school at Fergusonville, Delaware County, where he remained only a term and a-half. He was so intractable and vicious that he was at first separated from the other boys, and finally sent away. After remaining at home a short time he took a notion to go to sea. His father objected, but finally acquiesced, and furnished him an outfit. He was absent about a year, making a trip to Liverpool and several other ports. Returning, he was out of business for some time; he then obtained a situation as clerk in a real estate office, but soon after left it. His father then found a situation for him in the law office of Moody and Willis, corner of Broadway and Fulton-street. He exhibited no fitness for the place, and was in a short time discharged. His last attempt at business was as a clerk in the hardware store of Messrs.

Sullivan and Hyatt, in Platt-street; but he did not suit his employers, and lost his situation on the 1st of July last. During all the time he remained an inmate of his father's house, receiving the kindest and most careful attentions. He was, however, addicted to late hours, and his companions say to immoral practices. When the family went into the country last summer he accompanied them to Newburg, and remained with them until their return. At home he was at times pleasant towards his brothers and sisters, occasionally taking the little ones upon his knee and fondling them with much affection. At other times he was morose and revengeful, and exhibited an uncontrollable temper. He would not let the family know what he was about. He had a fondness for billiards, and it is supposed that he thus lost considerable money. During the religious excitement last winter he manifested much interest, and was admitted as a member on probation in the church to which his father and mother belonged; but he was finally dropped on account of his irregular habits. His father on Frank's account finally decided to embark once more in his former business, taking him in as a partner. A sum of money was deposited in the savings' bank to his credit, which he was told he would be permitted to draw on coming of age. Of ten dollars, which he had drawn on his father's bank-book on Tuesday morning, only forty cents. were found on his person after his miserable suicide. The slightest opposition would throw him into excitement, while his secretive propensity and obstinacy prevented his friends from tracing him to his haunts, or exercising any influence over him. . . . . There were never any indications of insanity in the family."

Have we here an example of homicidal mania, or of a premeditated and deliberately executed series of murderous attacks? The indiscriminate character of the attacks, the apparent absence of motive, and the mode in which the murderous acts were carried into effect, particularly when considered in relation with the ordinary disposition and habits of the assailer, seem to be inconsistent with the notion of sanity.

The second of the criminal events to which we have referred is a curious instance of juvenile crime:—

"The village of Bredbury, Cheshire, has been roused from its wonted quiet ness by an occurrence as extraordinary as it is unusual—the attempted murder of a girl 16 years of age by a boy 17 years old, through jealousy, who afterwards attempted to commit suicide. It appears that about nine o'clock on Tuesday evening last, as a girl named Fanny Bailey was returning to her home in Bredbury, from an evening school, she was met by a boy named William Bradshaw, 17 years of age [illegible] discharged a loaded pistol at her head and then ran away. The girl suffered a [illegible]ontusion in her left side, and her left hand was burnt by the explosion. The boy was found on Wednesday morning in his father's shippon, with his face covered with blood, arising, as it was found on examination, from his having discharged the contents of a pistol in his mouth. His wounds are described as of a dangerous character. On searching him a book was found in which was written:—'the cause of me doeing this Was because fany Baily Would Not Speak to me and i culd Not Live any longer so farewell Companions and Relations for ever But if fany Baily ever goes with any one els i will appear to her in my grave sute.' The case is in the hands of the police."—*Manchester Examiner*, Dec. 6.

A gradually increasing interest is being manifested in the so-called "intermediate" method of treating convicts, which has been comparatively recently introduced into the prison system of Ireland. The objects of this method, as stated by its introducer, Captain Crofton, are as follows:—

"The reformability of the generality of criminals has been admitted, after a laborious investigation by a Select Committee of the House of Commons in 1850, and their opinion has been corroborated by facts and figures in abundance. The acknowledged object of all prison treatment being so to direct its deterrent and reformatory course as shall best conduce to the required results—viz., the diminution of crime—it is considered that this result is obtained by a judicious combination of penal and reformatory treatment. The present system commencing with the deterrent, is followed by a course of penal and of reformatory discipline. The success of this system it is proposed to test previous to the release of any prisoner by the institution of a third stage, in which the reformatory element shall preponderate, as does the deterrent element in the first stage.

"The proposed stage of reformatory treatment places a prisoner where he can be assailed by temptations, and where the public will have an opportunity of judging of his reformation, of his industrious habits, and of his general fitness for employment. I firmly believe that it needs but satisfactory evidence of this fact to bring together the employer and those meriting and seeking employment; I firmly believe that this probationary stage, acting as a filterer between the prisons and the public, may be made the means of distinguishing the reformed convicts from the unreformed, before and after leaving their several places of confinement; and I believe the separation, operating as an important channel for amendment and prevention, will exercise an influence over the criminal population the value of which cannot be too highly appreciated."*

The beneficial effects which have followed from the adoption of this plan were fully set forth by the Earl of Carlisle at the last meeting of the National Association for the Promotion of Social Science, and they seem to warrant sanguine anticipations of the success of the system. Colonel Jebb, however, the Surveyor-General of Prisons in England, although approving of the abstract principles of the "intermediate system" of prison discipline, doubts whether it can be made applicable to male convicts in this country. He states that this system is in use in England among female convicts, the Fulham refuge for this class of prisoners being an intermediate prison; but notwithstanding the success of the system in that establishment, he conceives that the circumstances under which our male convicts are placed are unsuited to the adoption of intermediate prisons fo[illegible]m. The following are Colonel Jebb's conclusions:—

"First. The character of the convicts in this country and the circumstances differ so much from those in Ireland, that any plan for congregating them together under less control than is at present exercised, would not be calculated to render them more fit for discharge, or give the officers to whose care they might be consigned better, or even the same, opportunities of judging of their character, as those which exist at present.

"Secondly. That even if such objects could be promoted by removing selected convicts into separate small intermediate establishments, with diminished control and more voluntary action, the exhibition of convict discipline in such a form would impair the exemplary character and deterrent effects of a sentence of penal servitude, which, on all accounts, it is most essential to preserve as the most formidable of our secondary punishments.

"Thirdly. That however desirable it may be in a penal colony, and however successful in Ireland, it would be impossible in this country to carry out any

* "Report on the Discipline of the Convict Prisons for 1856 and 1857." By Colonel Jebb, C.B., pp. 92–93.

general superintendence over discharged prisoners by the police without interfering with the means of their obtaining employment, and thus a greater evil would be created than any good which could possibly follow.

"Fourthly. That the experience gained in Ireland of the advantages of assisting prisoners on discharge, fully confirms the views that have been frequently pressed upon the attention of the importance of such a measure, in order to secure the results of a good system of discipline.

Fifthly. That if such means could be systematically organized as proposed, p. 165, it would be very desirable to afford convicts some special information or instruction, in connexion with their future prospects, during the last few months of their confinement, not in separate intermediate establishments disconnected from the prisons, but in the stage of discipline which precedes discharge."*

Colonel Jebb conceives that the increased degree of association which would be necessitated among the convicts by the adoption of the intermediate system would be almost fatal to its success in England; his own experience, and that of the governors of most gaols, being conclusive as to the deteriorating effects of association among convicts. But Captain Crofton maintains that this experience was gained under different circumstances from those which now exist, and he remarks that if we cannot control our criminals in association, after their long discipline, we cannot expect the country to have confidence in their well-being. Colonel Jebb advances other objections to the unfitness of the system for England; but we do not clearly understand from his arguments how the system should prove successful, as it would appear to be, with female convicts here (among whom it might be supposed that the evils of greater association would be as operative as among the male convicts), and yet that the system should prove unfitted for the latter. It would seem, however, from Colonel Jebb's returns, that the per-centage of reformations in our present system of prison discipline among male convicts is at least equal to that in Captain Crofton's intermediate system.

The difference of feeling entertained in Ireland towards convicts as compared with that usually manifested in England, and the apparent impracticability of exercising a general superintendence over convicts discharged on the intermediate system in this country, without exercising a fatal effect upon the permanency of the circumstances which would confirm or perfect their reform, are difficulties which could not be easily overcome. Doubtless the suggestions of Colonel Jebb, contained in his fourth and fifth conclusions, are those best calculated for bringing about and securing the permanent reform of the criminal in England. The great thing required to perfect the existing means which we make use of for the reformation of criminals is a better disposition of the people at large to aid in the matter, by affording increased facilities for the employment of criminals after their discharge from prison, and thus preventing their relapse (at least from want of occupation) into criminal courses.

* "Colonel Jebb's Report," pp. 106-107.

